# Associations of Ready-to-Eat Cereal Consumption and Income With Dietary Outcomes: Results From the National Health and Nutrition Examination Survey 2015–2018

**DOI:** 10.3389/fnut.2022.816548

**Published:** 2022-03-29

**Authors:** Jessica Smith, Neha Jain, James Normington, Norton Holschuh, Yong Zhu

**Affiliations:** ^1^Bell Institute of Health and Nutrition, General Mills, Golden Valley, MN, United States; ^2^Statistics and Data Science, General Mills, Mississauga, ON, Canada; ^3^Statistics and Data Science, General Mills, Golden Valley, MN, United States

**Keywords:** dietary outcomes, diet quality, ready-to-eat breakfast cereals, income, poverty-to-income ratio, food groups

## Abstract

**Background:**

Ready-to-eat (RTE) cereal has been associated with higher diet quality but it is not known if this association differs by income.

**Objective:**

To investigate the association of RTE cereal with dietary outcomes in a US population stratified by income [measured using the poverty-to-income ratio (PIR)].

**Methods:**

Data from children 2–18 years (*n* = 5,028) and adults 19 years and older (*n* = 9,813) with 24-h dietary recalls from the cross-sectional, US nationally-representative 2015–2016 and 2017–2018 National Health and Nutrition Examination Surveys (NHANES) were used in a multivariable linear model that included cereal eating status (based on day 1 24 h dietary recall), PIR category (Low-PIR <1.85; Mid-PIR 1.85–3.50; High-PIR >3.50) and their interaction. PIR is based on the ratio of the family household income to the poverty level set by the US Department of Health and Human Services and higher PIR values indicate higher household income.

**Results:**

For children, there were positive associations between RTE cereal consumption and nutrient (e.g., iron, calcium, fiber, potassium and vitamin D, *p* < 0.001) and food group (e.g., whole grain and dairy, *p* < 0.001) intake and 2015-HEI (*p* < 0.0001) but no association with PIR or RTE cereal-PIR interaction. For adults, PIR category was positively associated with the intake of nutrients (e.g., fiber, magnesium, potassium, and vitamin C, *p* < 0.001) as was RTE cereal consumption (e.g., fiber, calcium, vitamin D, potassium, vitamin B_12_, among others, *p* < 0.001). Both PIR and RTE cereal were positively associated with whole grain, dairy, and fruit (*p* < 0.001) and 2015-HEI (*p* < 0.0001) for adults. We also found a significant interaction between PIR and RTE cereal for adults for iron, phosphorus, B vitamins, and dairy (*p* < 0.001). RTE cereal contributed to one quarter or more of daily intake, across all age and PIR groups, for several B vitamins, iron, zinc, and whole grains. Added sugar intake was not associated with RTE cereal consumption in either children or adults.

**Conclusion:**

RTE cereal was associated with improved dietary outcomes, including increased intake of under-consumed nutrients, increased intake of recommended food groups, and higher diet quality, at all income levels. This work can help inform future dietary recommendations.

## Introduction

Ready-to-eat (RTE) cereal is a staple breakfast food with approximately one-fifth of Americans consuming RTE cereal on any given day (1, 2). RTE cereal has been shown to be an important food nutritionally by encouraging milk consumption, contributing several key under-consumed dietary components including whole grains, fiber, iron, folate, vitamin B12, and vitamin D and being associated with overall higher diet quality in the US (1–3) and other countries (4–8). It has long been relied on as a pragmatic and affordable food that can help support dietary quality and remains an important part of several federal feeding programs.

Based on data from the 2019 US census, 10.5% of all Americans, and 14.4% of children younger than 18 were living in households with income below the poverty threshold (9). The poverty-to-income ratio (PIR) is a measure of a household's income compared to the poverty threshold set by the US government. Social determinants of health, including household income, have been associated with numerous health outcomes and this relationship may be mediated by diet quality. Access to affordable, acceptable foods that promote a high-quality diet may help to offset some of the negative health consequences of lower income status. However, there is limited information available on how specific food categories, such as RTE cereal, are associated with dietary outcomes at different income levels. Understanding the associations between income, specific foods and dietary outcomes is, therefore, essential to providing compelling and pragmatic dietary guidance.

The aim of this study was to investigate the association of RTE cereal, income, measured using PIR, and their interaction with dietary outcomes, including nutrient and food group intake, diet quality and the contribution of RTE cereal to nutrient and food group intake, in American children and adults. We hypothesized that RTE cereal would be associated with higher intake of key nutrients and food groups and higher diet quality at all income levels and that there would be a significant interaction between RTE cereal intake and income on these dietary outcomes.

## Methods

### Data Source and Study Participants

The National Health and Nutrition Examination Survey (NHANES) 2015–2016 (10) and 2017–2018 (11) and the Food Patterns Equivalents Database (FPED) 2015–2016 (12) and 2017–2018 (13) data were combined for these analyses. Participants aged 2 years and older, excluding women 20–44 years and older who were pregnant and lactating, with reliable (defined by NHANES) dietary data were included.

NHANES is a nationally representative survey of the US non-institutionalized population that is collected by the National Center for Health Statistics and the Centers for Disease Control and Prevention on an ongoing basis in 2-year cycles. NHANES collects demographic, dietary and health variables *via* an in-person examination and questionnaire. Dietary information was collected by a trained dietary interviewer using a 24-h dietary recall following a validated multi-pass methodology. Dietary information from children under 6 years of age was gathered using proxy interviews without the child present; for children 6–9 years of age, proxy interviews were conducted with the child present; and children 9–11 years provided information on their dietary intake with the assistance of an adult familiar with their intake. This study used de-identified publicly available data not requiring ethical review.

### Ready-to-Eat Cereal Consumption and Poverty-to-Income Ratio

RTE cereal consumption was defined according to the day 1 24-h dietary recall and was identified using the “Ready-to-Eat Cereal” category in the What We Eat in America food classification approach defined by United States Department of Agriculture (USDA) for use with NHANES. Participates that reported consuming any quantity of RTE cereal were defined as “Cereal Eaters” while all other participants were “Cereal Non-Eaters.” PIR is a pre-defined continuous variable in NHANES and is based on the ratio of the family household income to the poverty level set by the US Department of Health and Human Services. The higher the PIR value, the higher the household income with PIR values above 1 indicating household incomes above the poverty line and those at 1 or lower indicating household incomes at the poverty line or lower. We imputed missing PIR values (9% for children, 12% for adults) using a stepwise regression fitted separately for children and adults. We created three categories based on PIR: Low-PIR <1.85; Mid-PIR 1.85–3.50; and High-PIR >3.50. The lower cut-off is based on the eligibility criteria for some US federal food assistance programs including reduced price school meals and the Women, Infants and Children (WIC) program. The upper cut-off has been used previously to define higher-income (14).

We reported the following demographic characteristics of our stratified population: age (continuous); total energy intake (continuous); gender (dichotomous); ethnicity (categorical); overweight and obesity (categorical); smoking status (categorical); and household food security (categorical). Overweight and obesity, was defined in NHANES dataset based on adult BMI ≥25 kg/m^2^ or child BMI above the 85th percentile for age and sex according to Centers for Disease Control growth charts. Household food security is based on the number of affirmative responses to a food security survey administered as part of NHANES questionnaire (15).

### Study Outcomes

Daily food and beverage intake from the 24-h dietary recalls was converted to daily nutrient intakes in NHANES using the Food and Nutrient Database for Dietary Studies (FNDDS) 2015–2016 and 2017–2018 (16, 17). We reported results for 23 nutrients as the mean daily value. The FPED converts the foods and beverages in the FNDDS to the USDA Food Pattern food groups by disaggregating each individual food and beverage and summing the intake of each food group across the day. We reported the mean daily intake of 14 food groups and subgroups.

Diet quality was measured using the Healthy-Eating Index (HEI)-2015, developed by the National Cancer Institute, which measures how aligned an individual's daily dietary intake is with the recommendations of the 2015 Dietary Guidelines for Americans (18). Briefly, the HEI-2015 scores an individual's dietary intake, per 1,000 kcal, across 13 dimensions of nutrient and food group intake including nine adequacy components and 4 moderation components with a higher score indicating greater alignment with dietary recommendations and a maximum possible score of 100. Lastly, for RTE cereal eaters stratified by PIR categories, we calculated the percent contribution of RTE cereal to nutrient and food group intakes calculated as a ratio of the mean.

### Covariates

We adjusted our models for age (continuous), total energy intake (continuous), gender, and race/ethnicity. Race/ethnicity was self-selected by participants from the following categories: Mexican American; Other Hispanic; Non-Hispanic White; Non-Hispanic Black; Other Race including multi-racial.

### Statistical Analysis

SAS 9.3 (SAS Institute, Cary, NC, USA) was used for data analysis. NHANES 2015–2018 sample weight and SAS survey procedures were applied (19). We used a multivariable linear model to investigate the association between cereal eating status (cereal eaters vs. cereal non-eaters), PIR category (low-PIR, mid-PIR, high-PIR), and their interaction, adjusted for our covariates, with nutrient, food group, and diet quality outcomes. We also reported the p-values for a model that included cereal eating status, PIR as a continuous variable and their interaction in Table S1. We reported the lest-squared means for the six RTE cereal-PIR interaction terms from this model. We used a Bonferroni corrected *p*-value of 0.001 (0.05/36 nutrient and food group outcomes) for our nutrient and food group outcomes and a *p*-value of 0.004 (0.05/14 components & overall HEI score) for HEI-2015.

## Results

### Demographic Characteristics

Demographic characteristics of children and adults are reported in Table 1. Children were most likely to be in the low-PIR cereal non-eater group (27.4%) and least likely to be in the high-PIR cereal eater group (8.4%). For children, 35.7% of cereal eaters were in the low-PIR category, 33.3% in the mid-PIR category, and 28.5% in the high-PIR category (data not shown). Adults were also most likely to be in the high-PIR cereal non-eater group (34.3%) and least likely to be classified as mid-PIR cereal eaters (4.7%) (Table 1). For adults, 14.9, 15.4, and 16.0% for the low-, mid-, and high-PIR categories, respectively (data not shown).

**Table 1:** Demographic characteristics for American children and adults by ready-to-eat cereal consumption and poverty-to-income ratio, National Health and Nutrition Examination Survey, 2015–2018^a^.

	**Children 2–18 years**						**Adults 19 years and older**					
	**Low-PIR**		**Mid-PIR**		**High-PIR**		**Low-PIR**		**Mid-PIR**		**High-PIR**	
	**RTEC eaters**	**Non-eaters**	**RTEC eaters**	**Non-eaters**	**RTEC eaters**	**Non-eaters**	**RTEC eaters**	**Non-eaters**	**RTEC eaters**	**Non-eaters**	**RTEC eaters**	**Non-eaters**
*N* (% of population)	984 (17.0%)	1,767 (27.4%)	430 (9.1%)	854 (18.6%)	280 (8.4%)	712 (19.4%)	625 (5.0%)	3,616 (25.8%)	423 (4.7%)	2,389 (22.6%)	445 (7.6%)	2,314 (34.3%)
Age, mean ± SE, years	9.1 ± 0.2	10.5 ± 0.2	9.3 ± 0.3	10.5 ± 0.2	9.8 ± 0.4	10.6 ± 0.3	47.9 ± 1.2	44.5 ± 0.7	52.2 ± 1.7	48.1 ± 0.6	54.1 ± 1.0	49.0 ± 0.8
Gender, *n* (%) female	483 (49.1%)	931 (51.0%)	204 (46.9%)	418 (46.2%)	142 (52.6%)	348 (46.2%)	326 (52.1%)	1,891 (54.7%)	215 (50.4%)	1,190 (50.5%)	207 (51.0%)	1,134 (48.1%)
Energy intake, mean ± SE, kcal/day	1,840 ± 33	1,836 ± 35	1,916 ± 54	1,857 ± 34	1,839 ± 63	1,952 ± 41	2,292 ± 68	2,050 ± 24	2,286 ± 88	2,087 ± 36	2,128 ± 36	2,172 ± 28
Race/ethnicity, *n* (%) non-hispanic white	254 (36.3%)	389 (33.3%)	155 (53.7%)	311 (57.6%)	127 (71.8%)	324 (71.4%)	226 (52.3%)	1,032 (45.2%)	197 (70.3%)	757 (58.5%)	261 (86.0%)	942 (75.6%)
Overweight and Obesity, *n* (%)^b^	378 (37.8%)	733 (42.2%)	127 (29.5%)	331 (40.0%)	80 (26.9%)	186 (27.1%)	452 (70.0%)	2,636 (72.2%)	295 (66.9%)	1,178 (72.5%)	287 (67.1%)	1,671 (74.7%)
Smoking status, *n* (%) current smoker^c^	–	–	–	–	–	–	122 (20.8%)	941 (29.1%)	61 (15.9%)	433 (19.3%)	20 (3.5%)	238 (11.1%)
Household food security, *n* (%) full security^d^	337 (34.6%)	692 (38.3%)	260 (65.8%)	516 (64.9%)	253 (92.2%)	634 (92.4%)	259 (42.2%)	1,415 (41.9%)	303 (76.7%)	1,454 (63.5%)	411 (95.8%)	2,001 (90.3%)

a *Data are from the National Health and Nutrition Examination Survey (NHANES) 2015–2018 and are presented as mean ± standard error (SE) for continuous variables or n and percentage (%) for categorical variables, as indicated. Cereal eaters were defined as those that consumed any amount of ready-to-eat cereal on their day 1 24 h dietary recall. PIR bands were defined as Low-PIR <1.85; Mid-PIR 1.85–3.50; and High-PIR >3.50*.

b *Missing values for overweight and obesity prevalence ranged from 0.2% (n = 3) for high PIR children cereal eaters to 2% (n = 22) for low PIR children cereal eaters*.

c *Only reported in NHANES 2015–2018 for ages 12 years and older. There was 0.1% (n = 7) of data missing for low PIR adult cereal non-eaters and 0.1% (n = 2) of missing data for high PIR adult cereal non-eaters; all other categories had no missing values for smoking status*.

d *All PIR-cereal eating groups had some missing values for household full security ranging from 1.0% missing for high-PIR children cereal eaters to 6.0% for mid-PIR adult cereal non-eaters*.

Among children, cereal eaters in all PIR categories were on average slightly younger and, for adults, cereal eaters were slightly older. Total dietary energy intake ranged across groups from 1,836 to 1,952 kcal/day for children and from 2,050 to 2,292 kcal/day. The prevalence of children in the non-Hispanic white race/ethnicity category ranged from 33.3% for children and 45.2% for adults in the low-PIR cereal non-eater group to 71.8% for children and 86.0% for adults for the high-PIR cereal eater group. The prevalence of overweight and obesity among children ranged from 26.9% (high-PIR cereal eaters) to 42.2% (low-PIR cereal non-eaters) while for adults the prevalence ranged from 66.9% (mid-PIR cereal eaters) to 74.7% (high-PIR cereal non-eaters). Smoking status among adults ranged from 3.5% for the high-PIR cereal eaters to 29.1% for the low-PIR cereal non-eaters. Lastly, household food security for children was from 34.6% (low-PIR cereal eaters) to 92.4% (high-PIR cereal non-eaters) and for adults, it ranged from 41.9% (low-PIR cereal non-eaters) to 95.8% (high-PIR cereal eaters) (Table 1).

### Nutrient and Food Group Outcomes

For both children and adults, RTE cereal eating was associated with many of the nutrients and food groups investigated (Table 2 for children; Table 3 for adults). RTE cereal was associated with higher intake of carbohydrates, fiber, total sugar, calcium, iron, magnesium, phosphorus, potassium, zinc, folate, niacin, riboflavin, thiamine, vitamins A, B6, B12, C (adults only), and D, total dairy, fluid milk, total fruit (adults only), intact fruit (adults only) and whole grains. RTE cereal eating was also associated with lower intake for children and adults of total fat, saturated fat, selenium, sodium, refined grains (children only), total protein foods, and total meat, poultry, seafood, and egg intake (Tables 2, 3).

**Table 2:** Adjusted mean daily nutrient and food group intakes for American children by ready-to-eat cereal consumption and poverty-to-income ratio status, National Health and Nutrition Examination Survey, 2015–2018^a^.

	**Low-PIR**		**Mid-PIR**		**High-PIR**		***P*** **-values^b^**		
	**RTEC eaters**	**RTEC non-eaters**	**RTEC eaters**	**RTEC non-eaters**	**RTEC eaters**	**RTEC non-eaters**	**Cereal eating *p*-value**	**PIR category *p*-value**	Cereal eating*PIR category interaction *p*-value
Carbohydrate, g/d	254 ± 2	235 ± 1	254 ± 3	232 ± 2	256 ± 3	236 ± 3	<0.0001	0.65	0.68
Fiber, g/d	14.7 ± 0.2	13.3 ± 0.2	15.0 ± 0.5	14.1 ± 0.3	20.5 ± 0.8	16.7 ± 0.3	<0.0001	0.031	0.34
Total sugars, g/d	116 ± 2	103 ± 1	116 ± 3	101 ± 2	113 ± 3	103 ± 3	<0.0001	0.76	0.74
Protein, g/d	64.6 ± 0.9	66.1 ± 0.7	65.9 ± 1.4	67.2 ± 1.1	64.1 ± 1.5	66.9 ± 1.6	0.061	0.47	0.77
Total fat, g/d	68.2 ± 0.6	74.8 ± 0.5	68.1 ± 0.9	76.2 ± 0.9	68.4 ± 1.0	75.0 ± 0.8	<0.0001	0.72	0.47
Saturated fat, g/d	24.3 ± 0.3	25.5 ± 0.3	24.3 ± 0.5	26.3 ± 0.5	23.4 ± 0.6	25.5 ± 0.5	<0.0001	0.33	0.29
Calcium, mg/d	1,063 ± 19	889 ± 18	1,152 ± 41	902 ± 14	1,041 ± 28	892 ± 22	<0.0001	0.11	0.29
Iron, mg/d	18.5 ± 0.3	10.8 ± 0.2	18.0 ± 0.5	11.0 ± 0.2	18.3 ± 0.5	10.9 ± 0.2	<0.0001	0.86	0.59
Magnesium, mg/d	241 ± 2	219 ± 3	247 ± 5	223 ± 4	249 ± 7	230 ± 4	<0.0001	0.15	0.85
Phosphorus, mg/d	1,258 ± 14	1,191 ± 13	1,310 ± 28	1,212 ± 15	1,255 ± 31	1,201 ± 21	<0.0001	0.094	0.63
Potassium, mg/d	2,209 ± 29	2,023 ± 23	2,240 ± 46	2,025 ± 30	2,199 ± 43	2,100 ± 37	<0.0001	0.74	0.18
Selenium, μg/d	90.5 ± 1.5	95.9 ± 1.3	90.5 ± 2.2	98.1 ± 2.5	89.7 ± 2.8	95.5 ± 2.0	0.0001	0.78	0.83
Sodium, mg/d	2,813 ± 43	3,017 ± 24	2,804 ± 59	3,009 ± 33	2,786 ± 73	3,002 ± 57	0.0001	0.91	0.99
Zinc, mg/d	11.8 ± 0.2	8.3 ± 0.1	12.3 ± 0.3	8.3 ± 0.2	11.2 ± 0.4	8.1 ± 0.2	<0.0001	0.058	0.36
Folate, μg DFE/d^c^	746 ± 16	370 ± 8	708 ± 22	378 ± 7	692 ± 20	371 ± 11	<0.0001	0.16	0.23
Niacin, mg/d	25.2 ± 0.4	18.7 ± 0.3	24.9 ± 0.5	19.0 ± 0.4	24.4 ± 0.7	19.2 ± 0.6	<0.0001	0.93	0.35
Riboflavin, mg/d	2.29 ± 0.03	1.55 ± 0.03	2.31 ± 0.05	1.58 ± 0.03	2.16 ± 0.06	1.62 ± 0.03	<0.0001	0.35	0.03
Thiamine, mg/d	1.90 ± 0.03	1.31 ± 0.02	1.88 ± 0.04	1.33 ± 0.03	1.86 ± 0.06	1.32 ± 0.02	<0.0001	0.82	0.58
Vitamin A, μg RAE/d^d^	769 ± 15	459 ± 12	779 ± 28	485 ± 15	750 ± 35	514 ± 24	<0.0001	0.56	0.27
Vitamin B_6_, mg/d	2.29 ± 0.05	1.35 ± 0.03	2.23 ± 0.06	1.38 ± 0.03	2.10 ± 0.08	1.42 ± 0.05	<0.0001	0.50	0.060
Vitamin B_12_, μg/d	6.41 ± 0.14	3.47 ± 0.09	6.28 ± 0.16	3.43 ± 0.12	5.97 ± 0.30	3.16 ± 0.11	<0.0001	0.13	0.85
Vitamin C, mg/d	77.6 ± 3.2	71.2 ± 2.2	74.3 ± 6.8	67.5 ± 5.2	94.5 ± 8.1	79.0 ± 3.7	0.0070	0.068	0.47
Vitamin D, μg/d	7.40 ± 0.16	4.11 ± 0.15	7.17 ± 0.29	3.91 ± 0.14	6.41 ± 0.36	3.82 ± 0.15	<0.0001	0.024	0.26
Vitamin E, mg/d	7.22 ± 0.21	7.05 ± 0.16	6.94 ± 0.20	7.21 ± 0.19	8.54 ± 0.78	7.65 ± 0.27	0.37	0.13	0.16
Added sugars, tsp eq/d^e^	16.2 ± 0.4	15.5 ± 0.4	16.4 ± 0.6	14.8 ± 0.5	15.8 ± 0.6	14.4 ± 0.7	0.0065	0.39	0.54
Total dairy, cup eq/d	2.22 ± 0.05	1.66 ± 0.05	2.33 ± 0.10	1.69 ± 0.03	1.95 ± 0.10	1.57 ± 0.06	<0.0001	0.0074	0.21
Fluid milk, cup eq/d	1.56 ± 0.05	0.85 ± 0.05	1.44 ± 0.13	0.74 ± 0.06	1.29 ± 0.12	0.76 ± 0.04	<0.0001	0.10	0.36
Cheese and yogurt, cup eq/d	0.68 ± 0.03	0.78 ± 0.03	0.83 ± 0.08	0.85 ± 0.04	0.72 ± 0.06	0.74 ± 0.04	0.13	0.030	0.47
Total fruit, cup eq/d	1.12 ± 0.07	0.98 ± 0.05	1.05 ± 0.11	1.03 ± 0.08	1.28 ± 0.15	1.19 ± 0.06	0.14	0.12	0.76
Fruit juice, cup eq/d	0.41 ± 0.03	0.38 ± 0.02	0.36 ± 0.08	0.38 ± 0.06	0.35 ± 0.06	0.37 ± 0.03	0.83	0.41	0.75
Intact fruit, cup eq/d	0.71 ± 0.06	0.59 ± 0.04	0.69 ± 0.07	0.66 ± 0.04	0.93 ± 0.14	0.82 ± 0.06	0.10	0.049	0.69
Total grains, oz eq/d	6.60 ± 0.15	6.83 ± 0.12	6.59 ± 0.13	6.84 ± 0.12	7.02 ± 0.22	6.89 ± 0.15	0.31	0.33	0.41
Refined grains, oz eq/d	5.53 ± 0.16	6.20 ± 0.12	5.39 ± 0.14	6.06 ± 0.12	5.72 ± 0.22	6.15 ± 0.14	<0.0001	0.35	0.70
Whole grains, oz eq/d	1.07 ± 0.04	0.64 ± 0.05	1.21 ± 0.08	0.78 ± 0.05	1.31 ± 0.09	0.74 ± 0.07	<0.0001	0.015	0.58
Total protein foods, oz eq/d	3.79 ± 0.12	4.61 ± 0.14	3.75 ± 0.23	4.86 ± 0.18	3.94 ± 0.24	4.76 ± 0.21	<0.0001	0.68	0.71
Nuts, seeds and legumes, oz eq/d	0.65 ± 0.06	0.68 ± 0.05	0.75 ± 0.12	0.87 ± 0.10	0.76 ± 0.15	0.78 ± 0.09	0.41	0.076	0.88
Meat, poultry, seafood and eggs, oz eq/d	3.14 ± 0.13	3.93 ± 0.14	3.00 ± 0.21	3.99 ± 0.18	3.18 ± 0.25	3.97 ± 0.23	<0.0001	0.94	0.86
Total Vegetables, cup eq/d	0.84 ± 0.03	0.89 ± 0.02	0.85 ± 0.05	0.88 ± 0.03	0.80 ± 0.04	0.99 ± 0.04	0.0018	0.72	0.16

a *Data are from the National Health and Nutrition Examination Survey (NHANES) and Food Patterns Equivalent Database (FPED) 2015–2018 and are presented as mean ± standard error (SE). Cereal eaters were defined as those that consumed any amount of ready-to-eat cereal on their day 1 24 h dietary recall. PIR bands were defined as Low-PIR <1.85; Mid-PIR 1.85–3.50; and High-PIR >3.50*.

b *P-values were calculated using a multivariable linear model that included RTE cereal eating status (dichotomous), PIR status (categorical) and their interaction as the exposure and adjusted for age, gender, race/ethnicity and total energy intake. A p < 0.001 was considered statistically significant*.

c *DFE, Dietary folate equivalents 1 μg DFE = 1 μg naturally occurring food folate OR = 0.6 μg folic acid added in fortification*.

d *RAE, retinol activity equivalents 1 μg RAE = 1 μg retinol OR 12 μg dietary beta-carotene OR 24 μg dietary alpha-carotene or beta-cryptoxanthin*.

e *1 tsp eq. = 4.2 g of added sugar*.

**Table 3:** Adjusted mean daily nutrient and food group intakes and diet quality for American adults by ready-to-eat cereal consumption and poverty-to-income ratio status, National Health and Nutrition Examination Survey, 2015–2018^a^.

	**Low-PIR**		**Mid-PIR**		**High-PIR**		***P*** **-values^b^**		
	**RTEC eaters**	**RTEC non-eaters**	**RTEC eaters**	**RTEC non-eaters**	**RTEC eaters**	**RTEC non-eaters**	**Cereal eating *p*-value**	**PIR category *p*-value**	Cereal eating*PIR category interaction *p*-value
Carbohydrate, g/d	278 ± 3	250 ± 2	286 ± 5	243 ± 2	264 ± 4	234 ± 2	<0.0001	<0.0001	0.027
Fiber, g/d	19.6 ± 0.5	16.4 ± 0.3	20.5 ± 0.8	16.7 ± 0.3	24.0 ± 0.5	18.1 ± 0.3	<0.0001	<0.0001	0.025
Total sugars, g/d	120 ± 2	108 ± 2	130 ± 6	103 ± 2	109 ± 4	92 ± 2	<0.0001	0.0001	0.12
Protein, g/d	78.7 ± 1.5	79.1 ± 0.8	75.0 ± 1.8	82.3 ± 0.9	85.4 ± 1.9	84.2 ± 0.9	0.097	0.0001	0.0055
Total fat, g/d	75.7 ± 1.4	82.0 ± 0.6	72.7 ± 2.0	83.9 ± 0.8	77.6 ± 1.8	84.9 ± 0.8	<0.0001	0.099	0.14
Saturated fat, g/d	26.0 ± 0.5	25.9 ± 0.3	24.3 ± 0.7	27.1 ± 0.3	24.5 ± 0.7	26.7 ± 0.3	0.0002	0.72	0.0068
Calcium, mg/d	1163 ± 20	845 ± 8	1045 ± 31	887 ± 13	1128 ± 27	885 ± 12	<0.0001	0.14	0.0012
Iron, mg/d	22.7 ± 0.4	12.4 ± 0.1	22.5 ± 0.6	12.7 ± 0.1	20.2 ± 0.5	12.9 ± 0.1	<0.0001	0.0011	0.0002
Magnesium, mg/d	322 ± 6	283 ± 4	318 ± 7	298 ± 5	366 ± 9	314 ± 4	<0.0001	<0.0001	0.016
Phosphorus, mg/d	1,476 ± 26	1,300 ± 10	1,385 ± 22	1,337 ± 13	1,519 ± 26	1,359 ± 11	<0.0001	0.0005	0.0006
Potassium, mg/d	2,812 ± 51	2,467 ± 23	2,720 ± 71	2,545 ± 25	3,034 ± 52	2,658 ± 28	<0.0001	<0.0001	0.089
Selenium, μg/d	109 ± 4	115 ± 1	100 ± 3	117 ± 1	116 ± 3	118 ± 1	0.0004	0.014	0.0055
Sodium, mg/d	3,348 ± 50	3,457 ± 41	3,121 ± 60	3,520 ± 34	3,315 ± 80	3,563 ± 40	<0.0001	0.13	0.0011
Zinc, mg/d	13.5 ± 0.3	10.1 ± 0.1	13.2 ± 0.3	10.4 ± 0.1	13.7 ± 0.4	10.7 ± 0.1	<0.0001	0.31	0.45
Folate, μg/d DFE^c^	878 ± 27	434 ± 8	865 ± 27	433 ± 6	830 ± 22	452 ± 7	<0.0001	0.55	0.074
Niacin, mg/d	31.1 ± 0.9	24.3 ± 0.3	28.8 ± 0.7	24.9 ± 0.4	28.5 ± 0.6	25.1 ± 0.4	<0.0001	0.29	0.0083
Riboflavin, mg/d	2.73 ± 0.05	1.82 ± 0.02	2.47 ± 0.07	1.92 ± 0.03	2.41 ± 0.06	1.97 ± 0.03	<0.0001	0.098	<0.0001
Thiamine, mg/d	2.18 ± 0.04	1.46 ± 0.02	2.05 ± 0.04	1.49 ± 0.02	1.91 ± 0.04	1.51 ± 0.02	<0.0001	0.016	<0.0001
Vitamin A, μg/d RAE^d^	918 ± 31	498 ± 13	935 ± 78	569 ± 17	913 ± 50	605 ± 20	<0.0001	0.21	0.19
Vitamin B_6_ mg/d	3.07 ± 0.11	1.95 ± 0.06	2.67 ± 0.09	1.93 ± 0.05	2.793 ± 0.07	2.00 ± 0.06	<0.0001	0.10	0.0078
Vitamin B_12_, μg	8.22 ± 0.59	4.12 ± 0.11	6.48 ± 0.26	4.18 ± 0.13	6.80 ± 0.30	4.22 ± 0.13	<0.0001	0.12	0.031
Vitamin C, mg/d	85.5 ± 4.5	79.7 ± 2.3	88.0 ± 5.4	77.1 ± 2.6	105.7 ± 4.9	89.4 ± 3.0	0.0006	0.0001	0.37
Vitamin D, μg/d	8.25 ± 0.24	3.68 ± 0.11	6.54 ± 0.35	3.91 ± 0.17	7.06 ± 0.50	3.98 ± 0.17	<0.0001	0.023	0.0022
Vitamin E, mg/d	8.29 ± 0.40	8.47 ± 0.17	9.15 ± 0.42	8.70 ± 0.21	10.80 ± 0.79	9.53 ± 0.18	0.11	0.0010	0.20
Added sugars, tsp eq/d^e^	16.1 ± 0.6	17.6 ± 0.5	19.6 ± 1.5	16.2 ± 0.4	13.1 ± 0.8	13.2 ± 0.5	0.42	<0.0001	0.016
Total dairy, cup eq/d	2.10 ± 0.05	1.14 ± 0.02	1.76 ± 0.08	1.22 ± 0.04	1.74 ± 0.08	1.19 ± 0.04	<0.0001	0.0004	<0.0001
Fluid milk, cup eq/d	1.60 ± 0.10	0.42 ± 0.03	1.17 ± 0.09	0.46 ± 0.04	0.89 ± 0.10	0.40 ± 0.04	<0.0001	0.0001	0.0006
Cheese and yogurt, cup eq/d	0.560 ± 0.065	0.643 ± 0.020	0.620 ± 0.065	0.678 ± 0.027	0.649 ± 0.052	0.691 ± 0.031	0.23	0.0030	0.75
Total fruit, cup eq/d	1.18 ± 0.08	0.91 ± 0.04	1.22 ± 0.09	1.97 ± 0.04	1.56 ± 0.09	1.06 ± 0.05	<0.0001	0.0009	0.13
Fruit juice, cup eq/d	0.32 ± 0.05	0.27 ± 0.02	0.29 ± 0.05	0.24 ± 0.02	0.31 ± 0.04	0.24 ± 0.02	0.033	0.79	0.96
Intact fruit, cup eq/d	0.86 ± 0.07	0.64 ± 0.03	0.92 ± 0.08	0.73 ± 0.03	1.25 ± 0.10	0.81 ± 0.04	<0.0001	0.0004	0.14
Total grains oz eq/d	7.37 ± 0.2	6.71 ± 0.1	6.88 ± 0.1	6.60 ± 0.1	6.71 ±, 0.2	6.57 ± 0.1	0.012	0.0037	0.20
Refined grains, oz eq/d	5.96 ± 0.18	6.08 ± 0.11	5.44 ± 0.14	5.87 ± 0.09	5.23 ± 0.17	5.80 ± 0.11	0.022	0.0004	0.25
Whole grains, oz eq/d	1.41 ± 0.10	0.64 ± 0.04	1.44 ± 0.11	0.73 ± 0.05	1.49 ± 0.08	0.77 ± 0.04	<0.0001	0.33	0.90
Total protein foods, oz eq/d	5.53 ± 0.26	6.99 ± 0.12	5.48 ± 0.31	7.12 ± 0.13	7.02 ± 0.23	7.39 ± 0.15	<0.0001	<0.0001	0.0016
Nuts and seeds and legumes, oz eq/d	1.11 ± 0.11	1.37 ± 0.09	1.28 ± 0.17	1.30 ± 0.09	2.12 ± 0.24	1.58 ± 0.09	0.35	0.005	0.11
Meat, poultry, seafood and eggs, oz eq/d	4.42 ± 0.27	5.62 ± 0.11	4.19 ± 0.21	5.82 ± 0.10	4.90 ± 0.23	5.81 ± 0.13	<0.0001	0.13	0.12
Total vegetables cup, eq/d	1.27 ± 0.08	1.44 ± 0.03	1.43 ± 0.12	1.50 ± 0.04	1.65 ± 0.07	1.71 ± 0.06	0.099	<0.0001	0.53

a *Data are from the National Health and Nutrition Examination Survey (NHANES) and Food Patterns Equivalent Database (FPED) 2015–2018 and are presented as mean ± standard error (SE). Cereal eaters were defined as those that consumed any amount of ready-to-eat cereal on their day 1 24 h dietary recall. PIR bands were defined as Low-PIR <1.85; Mid-PIR 1.85–3.50; and High-PIR >3.50*.

b *P-values were calculated using a multivariable linear model that included RTE cereal eating status (dichotomous), PIR status (categorical) and their interaction as the exposure and adjusted for age, gender, race/ethnicity and total energy intake. A p < 0.001 was considered statistically significant*.

c *DFE, Dietary folate equivalents 1 μg DFE = 1 μg naturally occurring food folate OR = 0.6 μg folic acid added in fortification*.

d *RAE, retinol activity equivalents 1 μg RAE = 1 μg retinol OR 12 μg dietary beta-carotene OR 24 μg dietary alpha-carotene or beta-cryptoxanthin*.

e *1 tsp eq. = 4.2 g of added sugar*.

For children, PIR category and RTE cereal-PIR interaction were not significant for any nutrients or food groups (Table 3). Results were similar when PIR was included as a continuous variable (Table S1). For adults, there were negative associations with PIR for carbohydrates, total sugars, added sugar, and refined grain and positive associations for fiber, protein, magnesium, phosphorus, potassium, vitamin C, fluid milk, total fruit, intact fruit, and total vegetable intake. There were significant interactions between RTE cereal eating and PIR category among adults for iron, phosphorus, riboflavin, thiamine, total dairy, and fluid milk intake (Table 3).

### Diet Quality

RTE cereal consumption was significantly associated with higher diet quality for children and adults. For adults, but not children, PIR was positively associated with HEI (i.e., a higher PIR, meaning higher household income, was associated with better diet quality) and the RTE cereal-PIR interaction were also associated with HEI-2015, with larger gaps observed between cereal eaters and non-eaters at the mid-PIR and high-PIR categories compared to the low-PIR group (Table 4). For the HEI-2015 subcomponents, RTE cereal consumption, for both children and adults, was associated with a higher score (i.e., more aligned with dietary guidance) for the whole grains (*p* < 0.0001) total dairy (*p* < 0.0001), whole fruit (adults only, *p* < 0.0001), sodium (*p* ≤ 0.0001), refined grains (*p* ≤ 0.0006), and saturated fat (*p* ≤ 0.0003) components (Table 4). RTE cereal eating was also associated with lower scores (less aligned with guidelines) for the total protein foods (*p* ≤ 0.0003), fatty acids (*p* ≤ 0.0001), and added sugar (children only, *p* = 0.0024) components (Table 4).

**Table 4:** Healthy Eating Index total score and subcomponents for children and adults by ready-to-eat cereal consumption and poverty-to-income ratio status, National Health and Nutrition Examination Survey, 2015–2018^a^.

**HEI component (maximum score)**		**Low-PIR**		**Mid-PIR**		**High-PIR**		***P*** **-value^b^**		
		**RTEC eaters**	**RTEC non-eaters**	**RTEC eaters**	**RTEC non-eaters**	**RTEC eaters**	**RTEC non-eaters**	**Cereal eating *p*-value**	**PIR category *p*-value**	**Interaction *p*-value**
Total vegetables (5)	Children	2.1 ± 0.1	2.2 ± 0.0	2.1 ± 0.1	2.2 ± 0.1	2.1 ± 0.1	2.3 ± 0.1	0.093	0.64	0.67
	Adults	2.7 ±0.1	3.0 ± 0.0	3.0 ± 0.1	3.1 ± 0.1	3.5 ± 0.1	3.3 ± 0.1	0.15	<0.0001	0.022
Greens and beans (5)	Children	1.0 ± 0.1	1.0 ± 0.1	0.8 ± 0.2	1.0 ± 0.1	1.8 ± 0.2	1.6 ± 0.1	0.77	0.11	0.14
	Adults	1.4 ± 0.1	1.7 ± 0.1	1.8 ± 0.2	1.6 ± 0.1	2.5 ± 0.2	2.1 ± 0.1	0.36	<0.0001	0.0057
Total fruit (5)	Children	5.0 ± 0.0	5.0 ± 0.0	5.0 ± 0.0	5.0 ± 0.0	5.0 ± 0.0	5.0 ± 0.0	0.36	0.60	0.64
	Adults	5.0 ± 0.0	5.0 ± 0.0	5.0 ± 0.0	5.0 ± 0.0	5.0 ± 0.0	5.0 ± 0.0	0.19	0.68	0.78
Whole fruit (5)	Children	2.3 ± 0.1	2.0 ± 0.1	2.5 ± 0.2	2.3 ± 0.1	2.8 ± 0.3	2.7 ± 0.1	0.10	0.0024	0.88
	Adults	4.0 ± 0.2	1.9 ± 0.1	2.7 ± 0.2	2.0 ± 0.1	3.5 ± 0.2	2.4 ± 0.1	<0.0001	<0.0001	0.019
Whole grain (10)	Children	3.7 ± 0.1	2.1 ± 0.1	4.1 ± 0.2	2.5 ± 0.1	4.2 ± 0.2	2.4 ± 0.2	<0.0001	0.0066	0.90
	Adults	4.0 ± 0.2	1.9 ± 0.1	4.3 ± 0.3	2.2 ± 0.1	4.5 ± 0.2	2.2 ± 0.1	<0.0001	0.028	0.88
Dairy (10)	Children	7.7 ± 0.1	5.7 ± 0.3	7.6 ± 0.2	6.1 ± 0.1	7.0 ± 0.3	5.7 ± 0.2	<0.0001	0.066	0.043
	Adults	6.6 ± 0.1	3.8 ± 0.1	6.0 ± 0.2	4.1 ± 0.1	6.0 ± 0.2	4.1 ± 0.1	<0.0001	0.28	0.0008
Total protein food (5)	Children	3.3 ± 0.1	3.7 ± 0.1	3.4 ± 0.1	3.8 ± 0.1	3.4 ± 0.2	3.8 ± 0.1	0.0001	0.65	0.94
	Adults	3.9 ± 0.1	4.3 ± 0.0	3.8 ± 0.1	4.3 ± 0.0	4.3 ± 0.1	4.4 ± 0.0	0.0003	<0.0001	0.0003
Seafood and plant protein (5)	Children	1.7 ± 0.1	1.5 ± 0.1	1.7 ± 0.2	1.8 ± 0.1	1.7 ± 0.2	1.9 ± 0.1	0.43	0.13	0.10
	Adults	2.1 ± 0.1	2.3 ± 0.1	2.5 ± 0.1	2.4 ± 0.1	3.3 ± 0.1	2.7 ± 0.1	0.057	<0.0001	0.0035
Fatty acids (10)	Children	3.5 ± 0.2	4.3 ± 0.1	3.4 ± 0.2	4.1 ± 0.2	3.9 ± 0.4	4.5 ± 0.2	0.0001	0.23	0.91
	Adults	4.2 ± 0.2	5.4 ± 0.1	4.3 ± 0.2	5.2 ± 0.1	4.8 ± 0.3	4.1 ± 0.1	<0.0001	0.049	0.062
Sodium (10)	Children	5.5 ± 0.2	4.7 ± 0.1	5.5 ± 0.3	4.8 ± 0.1	5.6 ± 0.3	5.0 ± 0.2	0.0001	0.53	0.93
	Adults	5.0 ± 0.2	4.5 ± 0.1	5.4 ± 0.2	4.2 ± 0.1	4.8 ± 0.3	4.1 ± 0.1	<0.0001	0.17	0.044
Refined grains (10)	Children	5.5 ± 0.2	4.7 ± 0.2	5.8 ± 0.2	4.8 ± 0.2	5.9 ± 0.3	5.1 ± 0.2	<0.0001	0.013	0.83
	Adults	5.9 ± 0.2	5.5 ± 0.1	6.6 ± 0.2	5.8 ± 0.1	6.8 ± 0.2	5.9 ± 0.1	0.0006	0.0001	0.17
Saturated fat (10)	Children	5.7 ± 0.2	5.0 ± 0.1	5.5 ± 0.2	4.7 ± 0.2	5.9 ± 0.3	5.1 ± 0.2	<0.0001	0.32	0.87
	Adults	6.1 ± 0.2	6.1 ± 0.1	6.6 ± 0.2	5.7 ± 0.1	6.6 ± 0.2	5.8 ± 0.1	0.0003	0.76	0.021
Added sugar (10)	Children	6.4 ± 0.1	6.5 ± 0.1	6.1 ± 0.2	6.8 ± 0.2	6.4 ± 0.2	6.9 ± 0.2	0.0024	0.60	0.26
	Adults	6.8 ± 0.1	6.6 ± 0.1	6.3 ± 0.3	6.8 ± 0.1	7.6 ± 0.3	7.6 ± 0.1	0.41	<0.0001	0.067
Total HEI-2015 score (100)	Children	53.2 ± 0.7	48.4 ± 0.5	53.6 ± 0.9	50.1 ± 0.1	54.5 ± 0.7	50.8 ± 0.7	<0.0001	0.066	0.42
	Adults	56.1 ± 0.8	52.0 ± 0.4	58.2 ± 0.8	52.4 ± 0.5	63.5 ± 1.0	55.1 ± 0.4	<0.0001	<0.0001	0.013

a *Data are from the Food Patterns Equivalent Database (FPED) 2015–2018 and are presented as mean ± standard error (SE). Cereal eaters were defined as those that consumed any amount of ready-to-eat cereal on their day 1 24 h dietary recall. PIR bands were defined as Low-PIR <1.85; Mid-PIR 1.85–3.50; and High-PIR >3.50*.

b *P-values were calculated using a multivariable linear model that included RTE cereal eating status (dichotomous), PIR status (categorical) and their interaction as the exposure and adjusted for age, gender, race/ethnicity and total energy intake. A p < 0.004 was considered statistically significant*.

### Contribution of RTE Cereal to Daily Nutrient and Food Group Intake

RTE cereal contributed ~10% to daily energy intake across all age and PIR groups. RTE cereal contributed to one third or more of daily intake, across all age and PIR groups, for folate, iron, whole grains, and vitamins B6 and B12 (Table 5). Depending on age and PIR category, RTE cereal contributed to 14–21% of daily added sugar; 2–3% of saturated fat; and 6–9% of sodium intake (Table 5).

**Table 5:** Percent contribution of RTE cereal to daily intake of nutrients and food groups for RTE cereal eaters, stratified by age and Poverty-to-income (PIR) category^a^.

	**Children 2–18 years**			**Adults 19 years and older**		
	**Low-PIR *n* = 984**	**Mid-PIR *n* = 625**	**High-PIR *n* = 280**	**Low-PIR *n* = 625**	**Mid-PIR *n* =4 23**	**High-PIR *n* = 445**
Energy, %	9.9	9.0	9.2	9.8	9.8	9.4
Folate, %	58.0	53.8	50.2	56.2	56.2	51.0
Iron, %	52.9	49.7	49.7	53.2	52.4	45.4
Whole grains, %	45.5	44.3	50.9	51.3	55.0	60.5
Vitamin B_6_, %	45.1	43.0	40.2	39.7	39.2	34.7
Vitamin B_12_, %	41.7	38.5	38.8	36.9	38.3	34.5
Vitamin A, %	38.1	33.3	31.4	35.2	28.5	21.3
Thiamine, %	38.0	35.2	33.8	35.4	33.9	28.0
Niacin, %	37.6	34.7	32.3	32.7	31.0	24.6
Zinc, %	34.9	33.0	33.5	29.9	30.2	26.1
Riboflavin, %	31.2	27.7	28.1	27.6	26.5	20.1
Vitamin D, %	24.7	23.6	23.5	26.2	24.7	15.9
Added sugars, %	20.5	17.4	17.5	17.9	14.2	15.5
Fiber, %	17.2	17.5	18.9	21.7	22.8	22.0
Total grain, %	16.2	15.0	15.1	18.7	20.0	20.6
Carbohydrate, %	15.7	14.3	14.6	16.9	16.1	16.2
Vitamin C, %	14.7	13.6	11.1	13.0	11.9	7.5
Vitamin E, %	13.9	11.6	12.4	13.9	20.4	15.3
Total sugar, %	12.6	11.4	11.1	12.7	11.2	10.8
Refined grains, %	10.6	8.4	6.3	11.2	10.2	7.4
Magnesium, %	10.2	10.3	11.1	13.0	14.5	13.3
Sodium, %	9.0	8.0	7.0	8.0	7.4	5.7
Calcium, %	7.1	6.7	7.4	6.1	6.8	5.4
Phosphorus, %	6.8	6.8	8.1	8.8	10.5	10.0
Selenium, %	6.2	5.1	5.6	6.4	7.5	8.5
Protein, %	4.8	4.5	5.1	5.4	6.0	5.9
Potassium, %	4.7	5.1	5.6	6.6	7.0	6.6
Total fat, %	3.1	3.2	3.2	2.7	3.2	3.4
Nuts, seeds, and legumes, %	2.8	3.1	2.9	1.9	3.5	3.5
Saturated fat, %	2.6	2.5	2.6	2.0	2.1	1.9
Intact fruit, %	0.6	1.3	0.4	5.2	4.4	3.5
Total protein foods, %	0.5	0.6	0.6	0.4	0.8	1.1
Total fruit, %	0.4	0.9	0.3	3.7	3.4	2.9
Fluid milk, %	0.1	0.0	0.2	0.1	0.3	0.8
Total dairy, %	0.0	0.0	0.1	0.1	0.2	0.5
Fruit juice, %	0.0	0.0	0.0	0.0	0.0	0.0
Total vegetables, %	0.0	0.0	0.0	0.0	0.0	0.0
Cheese and yogurt, %	0.0	0.0	0.0	0.0	0.0	0.0
Meat, poultry, seafood, and eggs, %	0.0	0.0	0.0	0.0	0.0	0.0

a *Data are from the National Health and Nutrition Examination Survey (NHANES) and Food Patterns Equivalent Database (FPED) 2015–2018 and are presented as the percent contribution of nutrients and food groups from RTE cereal to daily intake calculated as a ratio of the means. Participants were RTE cereal eaters only and stratified according to age (children 2–18 years and adults 19 years and older), and poverty-to-income ratio (PIR). Cereal eaters were defined as those that consumed any amount of ready-to-eat cereal on their day 1 24 h dietary recall. PIR bands were defined as Low-PIR <1.85; Mid-PIR 1.85–3.50; and High-PIR >3.50. Nutrients and food groups (except for energy) are ranked in order from highest contribution to lowest contribution based on the children low-PIR group*.

## Discussion

This is the first study, to our knowledge, to show that RTE cereal is associated with higher consumption of under-consumed nutrients, recommended food groups and diet quality, for children and adults living in low-, mid-, and high-income households. It has been hypothesized that lower income status leads to deleterious dietary intakes, but we only found significant associations between PIR category and dietary outcomes for adults and not children. For adults, RTE cereal consumption may help offset some of the deleterious associations of lower-income status with nutrient intakes. These results are consistent with a previous study that also found income was associated with dietary outcomes in adults but not children (20). These associations may be due to the contribution of RTE cereal itself to dietary intakes—for example, among RTE cereal eaters, RTE cereal contributed to over a quarter of daily folate, iron, vitamin A, B vitamins, and vitamin D intake—or due to overall dietary patterns that are associated with RTE cereal consumption.

RTE cereal consumption, in both children and adults, was associated with higher intake of whole grains and fluid milk and, for adults, intact fruit consumption. RTE cereal is one of the top sources of whole grain in the US diet (21) and it has been shown that RTE cereal is eaten with fluid milk at over 80% of RTE cereal eating occasions (1, 2). Like our previous studies, we also found that RTE cereal consumption was associated with lower intake of meat, poultry and seafood (1, 2). A recently published meta-analysis found that dietary patterns in adults that were higher in whole grains, fruits, legumes, nuts, and seeds (among other components) and lower in red and processed meat were associated with lower all-cause mortality (22). Therefore, the food groups associated with RTE cereal may lead to a more favorable overall dietary pattern and potential beneficial health outcomes.

Despite numerous data, including the current study, showing overall beneficial dietary outcomes associated with RTE cereal intake, the sugar content of the RTE cereal category continues to be emphasized (21). We did find that total sugar intake was positively associated with RTE cereal consumption in children and adults. The increased total sugar intake among RTE cereal eaters may be due to the contribution of cereal itself (RTE cereal contributed 11–13% of daily total sugar intake across age and PIR groups) but also due to the higher intake of sugar containing food groups such as fruit and milk and possibly other foods. While RTE cereal did contribute to 14–18% of added sugar intake for adults and 18–21% of added sugar intake for children, added sugar was not associated with RTE cereal consumption. This could indicate compensation in added sugar intake may be taking place with non-RTE cereal eaters potentially consuming added sugar from other food categories. While RTE cereal contributes added sugar to the diet, it is also a nutrient dense food and, as this study has shown, is associated with beneficial dietary intake. Future research should further examine if not consuming RTE cereal is associated with compensatory increases in other dietary sources of added sugar and how that impacts overall dietary intake and quality.

As noted above, we found no gradient in dietary quality across our three income groups for children, but found that for adults, PIR was associated with diet quality. It is possible that children's diet quality may be more resilient in the face of food insecurity in part due to federal feeding programs such as WIC and the School Breakfast Program and National School Lunch Program. These supplemental feeding programs, run by the USDA, have nutrition standards, which have been periodically reviewed and strengthened over time to better align with the evidence-based recommendations within recent Dietary Guidelines for Americans. RTE cereal is included in the WIC package and is often served as part of subsidized school meals. A report from the USDA found that offering RTE cereal on every daily school breakfast menu was associated with a significantly higher HEI score (23) and that the children who consumed RTE cereal had higher whole grain intake and key nutrients including iron, calcium, vitamin D and fiber (24).

Diet cost, food preparation time, access to healthy foods, and nutrition knowledge are commonly cited barriers to healthy eating for those with lower incomes (25). The Thrifty Food Plan, recently released by the USDA, determined the market basket cost of a budget-conscious healthy dietary patterns and includes RTE cereal in its plan. Further work is needed that translate the recommendations within the Dietary Guidelines for Americans into pragmatic dietary advice around specific food categories with consideration to cost, accessibility, and/or ease of preparation for individuals and families facing barriers to healthy eating.

This study has several strengths including using nationally representative dietary intake data collected using rigorous methodology that is publicly available. Further, our analyses relied on detailed dietary intake collected using 24 h recalls through a rigorous validated multiple-pass approach. While 24 h dietary recalls cannot provide estimates of usual dietary intake for individuals, which would be needed studies examining health outcomes, they do provide reliable estimates of mean usual intake for populations (26).

Despite these strengths, several limitations that should be acknowledged. First, this study used a cross-sectional observational design which limits our ability to make causal inferences. However, because our outcomes are dietary related, we wouldn't expect reverse causation, or significant lag time between exposure and outcomes in this study. Second, this study relies on self-reported dietary intake, which is well-known to include systematic bias. It is possible there may be differences in dietary reporting by PIR status; however, we did not see significant differences in calorie intake across our PIR bands, suggesting that each group reported similar amounts of food intake. It also remains possible that there is residual confounding. We did not fully explore all the possible social determinants of health and there are important intersections between race/ethnicity, immigration status, education, childhood adversity, and income with dietary intake and health status.

Identifying nutrient dense affordable food choices to help support healthy dietary patterns could be a strategy to increase diet quality across all income levels. RTE cereal was associated with improved dietary outcomes, including increased intake of under-consumed nutrients, increased intake of recommended food groups, and higher diet quality, at all income levels. This work can help inform future dietary recommendation and nutrient policy and regulations, particularly those directed toward improving the dietary intakes of lower-income Americans.

## Data Availability Statement

Publicly available datasets were analyzed in this study. This data can be found here: https://wwwn.cdc.gov/nchs/nhanes/Default.aspx.

## Ethics Statement

The studies involving human participants were reviewed and approved by NCHS Research Ethics Review Board. The present study used publicly available NHANES data as secondary analysis, which is exempt from IRB review. Written informed consent to participate in the NHANES study was provided by the participants (for adults) or their legal guardian (for children).

## Author Contributions

JS and YZ designed research. NJ, JN, and NH analyzed the data and performed statistical analysis. JS wrote the article and had primary responsibility for final content. All authors have read and approved the final content.

## Funding

Financial support for this study was provided by General Mills Bell Institute of Health and Nutrition.

## Conflict of Interest

All authors are employees of General Mills, a manufacturer of several food categories, including ready-to-eat cereal. The authors declare that this study received funding from General Mills Bell Institute of Health and Nutrition. The funder had the following involvement in the study: study design, data analysis, interpretation of data, writing of this article, and decision to submit it for publication.

## Publisher's Note

All claims expressed in this article are solely those of the authors and do not necessarily represent those of their affiliated organizations, or those of the publisher, the editors and the reviewers. Any product that may be evaluated in this article, or claim that may be made by its manufacturer, is not guaranteed or endorsed by the publisher.
