# Supplementary material for: Associations of Ready-to-Eat Cereal Consumption and Income With Dietary Outcomes: Results From the National Health and Nutrition Examination Survey 2015–2018
Source: Front Nutr. 2022 Mar 29;9:816548. doi: 10.3389/fnut.2022.816548 (PMC9002128; doi:10.3389/fnut.2022.816548)
Supplement: Supplementary file 1 [file Table_1.DOCX]

**Supplementary Table**

Table S1. P values for an adjusted model that examined the association between ready-to-eat cereal intake, poverty-to-income ratio, and their interaction with nutrient intakes for American children and adults, National Health and Nutrition Examination Survey, 2015-2018^1^

|  | Children 2 – 18 years | | | Adults 19 years and older | | |
| --- | --- | --- | --- | --- | --- | --- |
|  | Cereal eating p value | PIR p value | Cereal Eating*PIR interaction p value | Cereal eating p value | PIR p value | Cereal Eating*PIR interaction p value |
| Carbohydrate | <0.0001 | 0.50 | 0.62 | <0.0001 | <0.0001 | 0.98 |
| Fibre | 0.062 | 0.0068 | 0.16 | 0.022 | <0.0001 | 0.007 |
| Total sugars | <0.0001 | 0.38 | 0.71 | 0.0068 | <0.0001 | 0.76 |
| Protein | 0.60 | 0.98 | 0.36 | 0.13 | <0.0001 | 0.29 |
| Total fat | <0.0001 | 0.93 | 0.87 | 0.0004 | 0.038 | 0.87 |
| Saturated fat | 0.044 | 0.39 | 0.56 | 0.96 | 0.54 | 0.053 |
| Calcium | <0.0001 | 0.88 | 0.80 | <0.0001 | 0.14 | 0.26 |
| Iron | <0.0001 | 0.91 | 0.54 | <0.0001 | 0.0034 | <0.0001 |
| Magnesium | 0.0005 | 0.044 | 0.73 | 0.092 | <0.0001 | 0.073 |
| Phosphorus | 0.0008 | 0.60 | 0.84 | 0.0005 | 0.0001 | 0.73 |
| Potassium | <0.0001 | 0.49 | 0.22 | 0.0014 | <0.0001 | 0.24 |
| Selenium | 0.073 | 0.82 | 0.66 | 0.026 | 0.0076 | 0.29 |
| Sodium | 0.0081 | 0.23 | 0.34 | 0.082 | 0.14 | 0.46 |
| Zinc | <0.0001 | 0.053 | 0.62 | <0.0001 | 0.059 | 0.46 |
| Folate | <0.0001 | 0.060 | 0.10 | <0.0001 | 0.75 | 0.077 |
| Niacin | <0.0001 | 0.56 | 0.098 | <0.0001 | 0.42 | 0.012 |
| Riboflavin | <0.0001 | 0.59 | 0.017 | <0.0001 | 0.37 | <0.0001 |
| Thiamine | <0.0001 | 0.77 | 0.36 | <0.0001 | 0.0045 | <0.0001 |
| Vitamin A | <0.0001 | 0.056 | 0.37 | <0.0001 | 0.041 | 0.20 |
| Vitamin B_6_ | <0.0001 | 0.33 | 0.031 | <0.0001 | 0.17 | 0.036 |
| Vitamin B_12_ | <0.0001 | 0.13 | 0.85 | <0.0001 | 0.12 | 0.031 |
| Vitamin C | 0.21 | 0.086 | 0.43 | 0.57 | 0.0002 | 0.17 |
| Vitamin D | <0.0001 | 0.0090 | 0.29 | <0.0001 | 0.24 | 0.0047 |
| Vitamin E | 0.40 | 0.056 | 0.24 | 0.17 | 0.0002 | 0.057 |

^1^ Data are from the National Health and Nutrition Examination Survey (NHANES) and Food Patterns Equivalent Database (FPED) 2015-2018 and are presented as p values that were calculated using a multivariable linear model that included RTE cereal eating status (dichotomous), PIR (continuous) and their interaction as the exposure and adjusted for age, gender, race/ethnicity and total energy intake. A p<0.001 was considered statistically significant. Cereal eaters were defined as those that consumed any amount of ready-to-eat cereal on their day 1 24 hr dietary recall. PIR was included as a continuous variable.
